# Impact of an Intergenerational Bonding Program on Dimensions of Health and Related Outcomes Among Older Adults: A Meta-Analysis

**DOI:** 10.7759/cureus.95366

**Published:** 2025-10-25

**Authors:** Monika Kankarwal, Neetu Kataria, Sachin Kumar, Thokchom Soniya Devi, Kusum Kumari, I.Ananda Hariharan, Lizy Sonia Benjamin, Vasantha C Kalyani

**Affiliations:** 1 Medical Surgical Nursing, All India Institute of Medical Sciences, Deoghar, Deoghar, IND; 2 Medical Surgical Nursing, All India Institute of Medical Sciences, Rishikesh, Rishikesh, IND; 3 Anesthesia, Hindu Rao Hospital, Delhi, IND; 4 Psychiatry Nursing, All India Institute of Medical Sciences, Deoghar, Deoghar, IND; 5 General Practice, Anna Medical College, Montagne Blanche, MUS; 6 Medical Surgical Nursing, King Khalid University, Abha, SAU

**Keywords:** dimensions of health, intergenerational bonding program, mental health, older adults, physical health, social health, well-being

## Abstract

Nowadays, with people living longer and family structures changing, older adults often experience loneliness and neglect. This study aimed to assess the impact of an intergenerational bonding program on dimensions of health and related outcomes among older adults. An extensive search was carried out between January 1, 2014, and October 4, 2023, using PubMed, ScienceDirect, Scopus, and Google Scholar. The Preferred Reporting Items for Systematic Reviews and Meta-Analyses (PRISMA) 2020 guideline was followed in this study. Heterogeneity was assessed through the I^2^ statistics by using a fixed-effect model, and the risk ratio was calculated. A total of 234 participants from the four trials were included in the meta-analysis. Out of the five outcomes, social health showed a significant improvement in the intergenerational program group as compared to the control group with a mean difference (MD) of -0.27 (95% CI -0.48 to -0.06; p=0.02* (*: statistically significant improvement)) from two studies without any significant heterogeneity (I^2^=0%; p=0.34). Although a pooled analysis of mental health favors the intergenerational program group with an MD of -0.10 (95% CI 0.24 to 0.03; p<0.14), it was not statistically significant. However, the remaining three outcomes, namely, physical health, well-being, and meaningfulness outcomes, did not favor either the intergenerational program group or the control group (p>0.05). Social health improved in the intergenerational program group. The study findings support a strong recommendation for the implementation of the intergenerational program to uplift the social relationships of older adults. The rest of the dimensions of health need to be further evaluated with large sample size studies.

## Introduction and background

Older adults aged 60 years and above are increasing steadily and gradually across the world. In 2023, 1.1 billion people in the world were aged 60 years or over as per the World Health Organization (WHO). With increasing life expectancy, aging is an emerging phenomenon, and aging care is the utmost priority [1]. Older adults are an integral part of society in many ways and a valuable resource for society. They act as an ideal mentor and play a major role in resolving disputes among family members by holding the family together in every circumstance and strengthening them. Older adults are like an immense ocean, full of knowledge, experiences, and wisdom, which acts as a guidepost for the younger generation [2]. India, known for its filial piety virtues, characterized by diversity and a wealth of cultural norms and practices, is now facing a chain of transformation from a joint family status to a nuclear family status due to the onset of modernization and globalization. This transition has weakened the traditional virtue of caring and providing support to older adults, affecting the intergenerational relationship with them [3,4]. Because of this, older adults face loneliness, social isolation, cognitive decline, and an increased risk of having chronic illnesses [5].

Fortunately, the easy solution for fixing these issues is intergenerational bonding, where older adults interact with young adults through planned and mutually beneficial activities/programs, making them feel connected to others and receive much-needed stimulation [6].

A recent study found that older adults participating in the Experience Corps program demonstrated greater generative desire and a stronger sense of generative achievement compared to those in the control group [7].** **Another longitudinal study conducted in Japan revealed that older adults who had undergone the individualized development plan (IDP) had significantly improved health-related quality of life (HRQOL) scores and decreased depression [8]. A study conducted to assess the effectiveness of the Intergenerational Mentor-Up (IMU) innovative program on older adults' e-healthy literacy and social isolation domain reported decreased social isolation and anxiety and increased confidence among them [9]. A review conducted to study intergenerational engagement and its effects on older adults' cognitive, social, and health outcomes found considerable variation in how such programs are designed. It recommended further research using diverse methods to enable broader implementation and more generalizable results [10]. 

To the best of our knowledge, no study has provided comprehensive findings that specifically address all aspects of health outcomes related to intergenerational bonding. Therefore, the authors concluded that there is an utmost need to conduct a meta-analysis to assess the impact of an intergenerational bonding program on dimensions of health and related outcomes among older adults in order to enable the generalization of the results among them and to develop an evidence-based, culturally appropriate intergenerational bonding program for them.

## Review

Materials and methods

**Figure 1:**
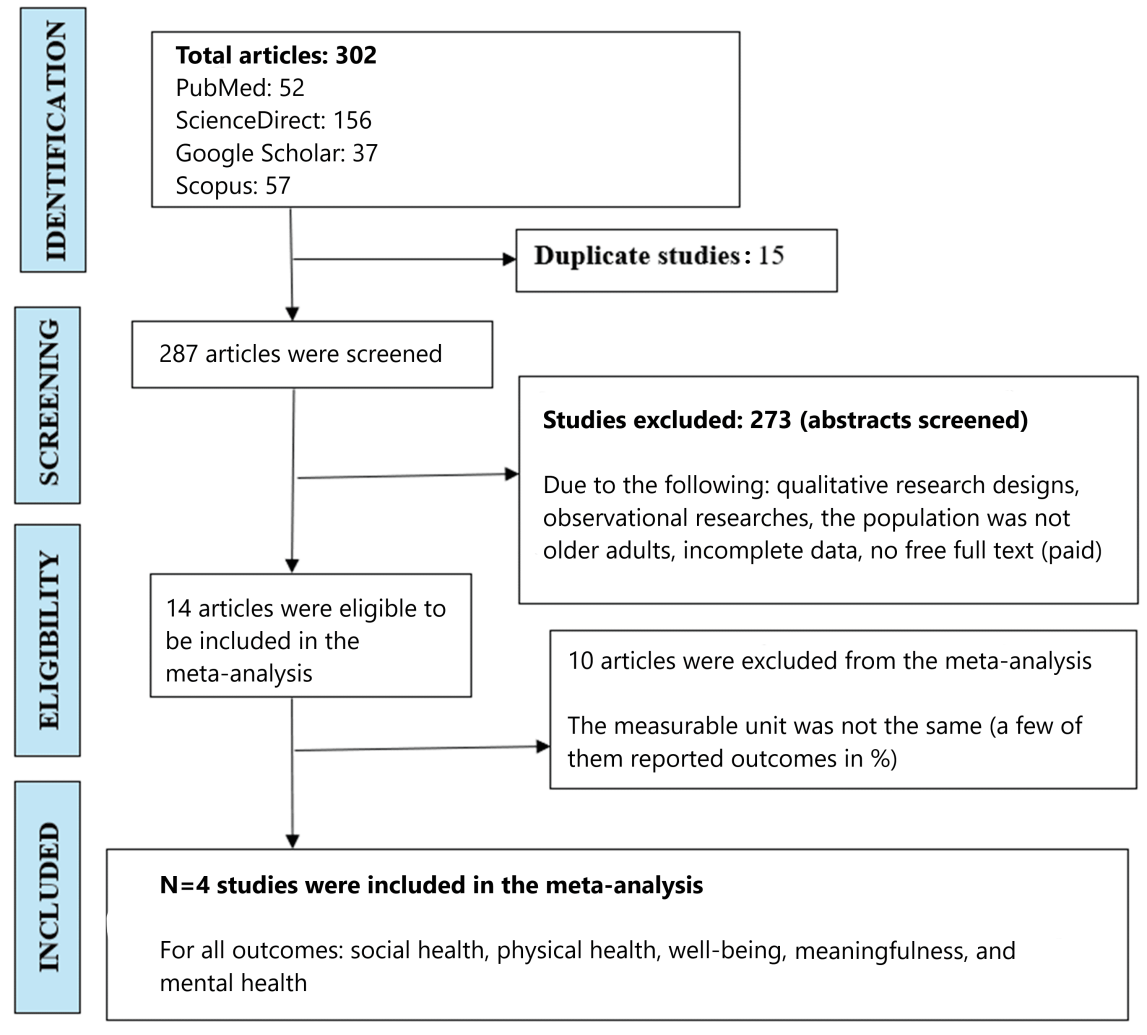
PRISMA flow diagram PRISMA: Preferred Reporting Items for Systematic Reviews and Meta-Analyses

We evaluated the effect of an intergenerational bonding program on the social health, physical health, well-being, mental health, and meaningfulness dimensions of older adults by adhering to the Preferred Reporting Items for Systematic Reviews and Meta-Analyses (PRISMA) 2020 guideline (Figure 1) [11]. The study is registered in the International Prospective Register of Systematic Reviews (PROSPERO) with ID no. CRD42023403997.

Data Sources and Selection Criteria

We conducted an online search trial on published research articles between January 1, 2014, and October 4, 2023, using PubMed, ScienceDirect, Scopus, and Google Scholar (refer to the Appendices for the search methodologies). Only English-language research was included. The search strategy followed the Population, Intervention, Control, and Outcomes (PICO) framework, where P denoted older adults aged 60 years and above, I an intergenerational bonding program, C no intervention or standard routine care or placebo, and O the effect of an intergenerational bonding program on the social health, physical health, well-being, mental health, and meaningfulness outcomes of older adults. The population "old age" and the intervention "intergeneration relations", "relations, intergeneration", and "intergeneration relation" were the Medical Subject Headings (MeSH) terms that were utilized. The comparison group was "no intervention/routine care", and the outcome variables were "social", "mental", "meaningfulness", physical health", and "well-being". We looked for any more trials in the lists of references from the papers included.

Study Selection

After conducting an independent screening of article titles and abstracts, two independent authors (MK and NK) identified online and published research articles. Online published randomized controlled trials (RCTs) and quasi-experimental studies in the English language were screened from January 2014 to October 2023 for this meta-analysis. The studies include participants 60 years and above, providing data on the potential change of baseline scores to a later timepoint after intervening with an intergenerational bonding program on at least one or all outcomes together within the social health, mental health, meaningfulness, physical health, and well-being outcome variables. The exclusion criteria included studies with qualitative research designs, observational research, populations other than older adults, incomplete data, and non-availability of free full text (paid).

Outcome Variables

The outcome variables from the included studies, such as study characteristics and participant profiles, were examined by both independent authors (MK and NK). The main outcome of the meta-analysis was to evaluate the impact of intergenerational bonding programs on dimensions of health, namely, social health, mental health, meaningfulness, physical health, and well-being, among older adults.

Data Extraction

Data extraction of the selected studies was performed by two independent authors (MK and NK). Any disagreements regarding eligibility were resolved by discussion between the authors. After selecting each study, data were extracted and organized to include the study title, author, year of publication, number of participants, details of experimental and control groups, mean age, percentage of male participants, interventions for the experimental group, and conditions for the control group. Any required clarifications were obtained by contacting the authors through email.

Risk of Bias and Quality Assessment

**Figure 2:**
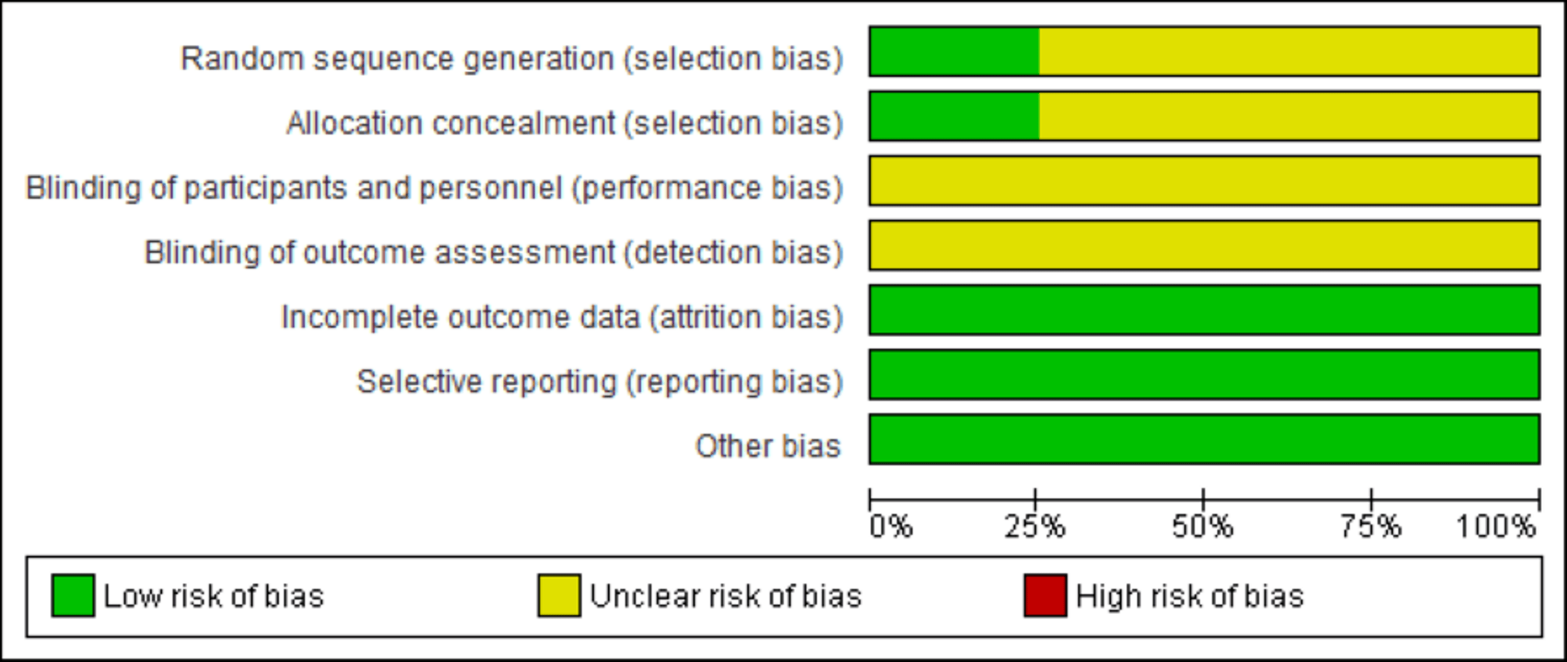
Risk of bias graph

**Figure 3:**
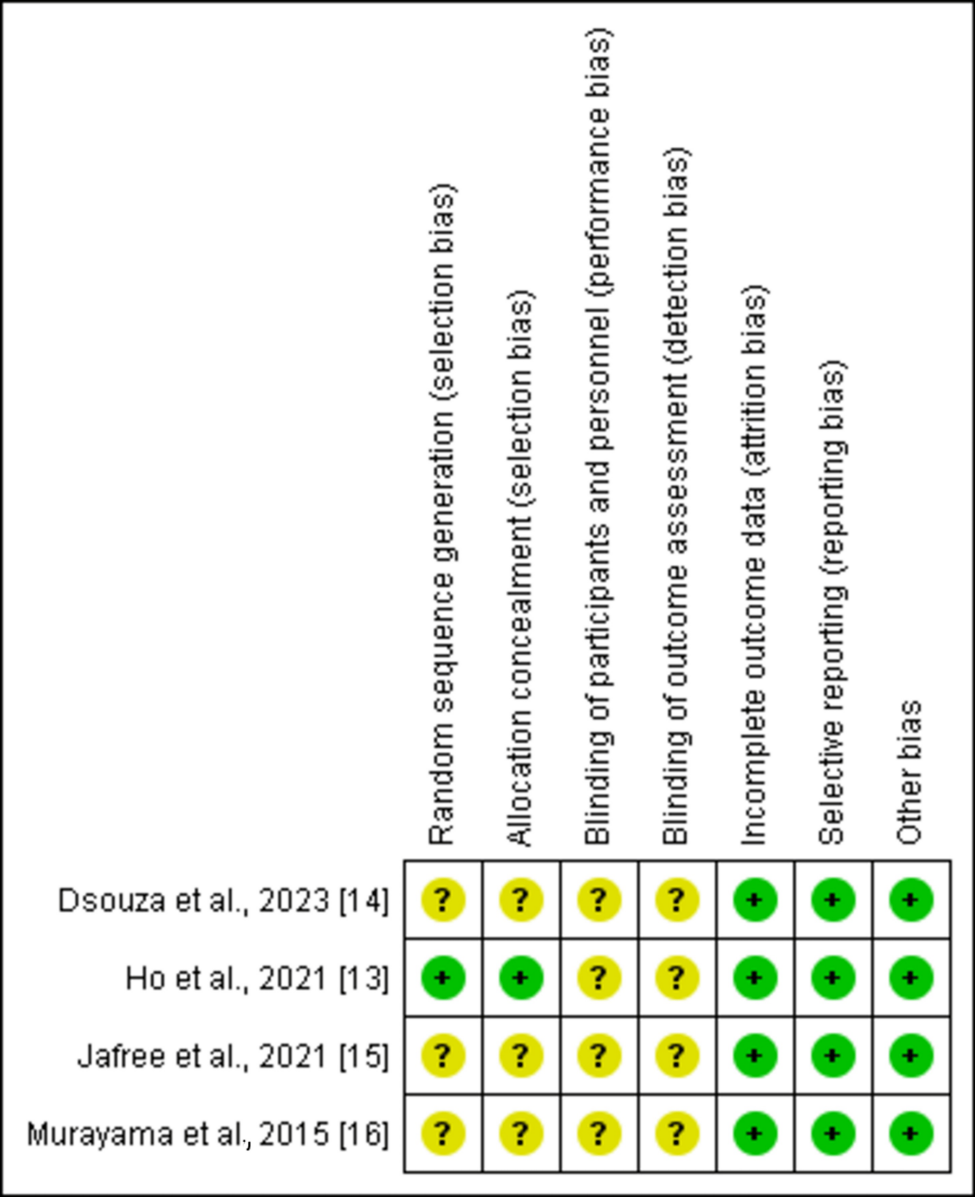
Risk of bias summary

Authors MK and NK performed the risk of bias assessment independently. The Cochrane risk of bias assessment tool was used [12]. Figures 2-3 present the risk of bias assessment, with Figure 2 showing the overall risk of bias graph and Figure 3 providing a summary for each selected study, covering selection, performance, detection, attrition, reporting, and other potential biases.

All studies were labeled as low, high, and unclear risk for each component. All disagreements between the two independent authors were resolved by consensus. The randomized sequence methods and allocation concealment were properly described in a single study [13], whereas three studies showed unclear status [14-16]. A double-blind design and blinding of outcome assessors among all four studies showed an unclear status [13-16]. Data outcome and selective reporting of study data were clearly described in all studies [13-16]. 

Data Analysis

The final statistical analysis was carried out according to the protocol of the latest edition of the Cochrane Handbook for Systematic Reviews of Interventions and by using the RevMan 5.4 software package (The Cochrane Collaboration, London, England, United Kingdom).

Quantitative data of outcome variables, social health, mental health, physical health, well-being, and meaningfulness, were in the form of mean difference (MD) with 95% CI. Heterogeneity was assessed by using the I^2^ statistics for treatment effects among selected trials, which was assessed using the chi-squared test. An I^2^ of 0% was categorized as "no heterogeneity", 50% as "minimal heterogeneity", and >50% as "substantial heterogeneity". The final meta-analysis was conducted using a fixed-effect model, with statistical significance set at p≤0.05 and an I² value ≤50%. Nonoverlapping confidence intervals in the forest plot were interpreted as indicating heterogeneity. All p-values were statistically significant at ≤0.05 at two-tailed. Potential publication bias was evaluated by constructing a funnel plot of the MD against the standard error to examine the primary outcomes related to social health. To assess the influence of RCTs, a sensitivity analysis was performed, and the trials with enormous effects that were causing heterogeneity in the results were excluded, one by one.

Results

Selection and Characteristics of the Studied Trials

The PRISMA 2020 guideline [11] was used for drawing the flow diagram, which reported, screened, excluded, and finally included articles through the search strategy. Using the PICO format, 302 studies were included (PubMed 52, ScienceDirect 156, Google Scholar 37, Scopus 57), and 273 studies were excluded (as shown in Figure 1). Accordingly, four full-text interventional studies that satisfied all the eligibility criteria for this meta-analysis were ultimately included, as presented in Table 1.

**Table 1:** Characteristics of the included studies NM: not mentioned in mean±SD

S. no.	Author, year	Exp./Cont. gp (f)	Age Exp./Cont. gp (mean±SD)	Male Exp./Cont. gp (f)	Experimental group	Settings
1	Ho et al., 2021 [13]	17/17	72.6±7.5/73.6±5.5	3/3	Intervention contents such as weekly themes, schedules, and art activities were jointly developed by community partners, museum representatives, artists, and the research team	UK
2	Dsouza et al., 2023 [14]	41/43	NM	10/28	Two-day (8 hours daily) program including various activities. The children interacted with older adults in the experimental group once a week for 12 weeks for about 45-60 minutes	India
3	Jafree et al., 2021[15]	18/18	68.4±6.06/68.4±6.06	13/13	Intergenerational Program of Learning	Pakistan
4	Murayama et al., 2015 [16]	26/54	68.8±4.1/69.3±3.4	2/11	Research of Productivity by Intergenerational Sympathy (REPRINTS) program	Japan

A total of 234 participants from the four trials belong to the older adult age group, along with their children or adolescents. Two trials had complete data on all outcome variables, which are physical health, social health, mental health, well-being, and meaningfulness, whereas a single study [16] had data on mental health and meaningfulness outcome variables. Dsouza et al. [14] had outcome data on the meaningfulness variable. Out of the four trials, a single trial [13] was an RCT, while three trials [14-16] were quasi-experimental studies. 

A sensitivity analysis evaluating the impact of intergenerational bonding programs on well-being, conducted by excluding the study by Dsouza et al. [14], revealed a notable change in heterogeneity, with an MD of 0.32 (95% CI: 0.06-0.58; p<0.61) and heterogeneity values of I²=26% and p<0.26 (Table 2). Hence, one trial by Dsouza et al. [14] was removed, and Ho et al. [13] and Jafree et al. [15] were included in the pooled analysis.

**Table 2:** Sensitivity analysis on the well-being outcome among older adults *: statistically significant improvement/difference MD: mean difference; CI: confidence interval; P_s_: p-value for significance; I^2^: study heterogeneity; P_H_: p-value for heterogeneity

The omitted trial: author, year	MD (95% CI)	P_S_	I^2^	P_H_
Dsouza et al., 2023 [14]	13.01 [6.46, 19.57]	0.0001*	99%	0.0001*
Ho et al., 2021 [13]	Included	-	-	-
Jafree et al., 2021[15]				
Overall effect	0.32 [0.06, 0.58]	0.61	26%	0.26

A sensitivity analysis examining the effect of intergenerational bonding programs on meaningfulness, conducted by excluding the study by Ho et al. [13], showed a significant change in heterogeneity, with an MD of 0.86 (95% CI: 0.19-1.53; p<0.01* (*: statistically significant improvement)) and heterogeneity values of I²=18% and p<0.27 (Table 3). Hence, one trial by Ho et al. [13] was removed, and Murayama et al. [16] and Jafree et al. [15] were included in the pooled analysis.

**Table 3:** Sensitivity analysis on the meaningfulness outcome among older adults *: statistically significant improvement/difference MD: mean difference; CI: confidence interval; P_s_: p-value for significance; I^2^: study heterogeneity; P_H_: p-value for heterogeneity

The omitted trial: author, year	MD (95% CI)	P_S_	I^2^	P_H_
Ho et al., 2021 [13]	0.06 [-0.16, 0.29]	0.59	73%	0.03
Murayama et al., 2015 [16]	Included	-	-	-
Jafree et al., 2021[15]				
Overall effect	0.86 [0.19, 1.53]	0.01*	18%	0.27

**Figure 4:**
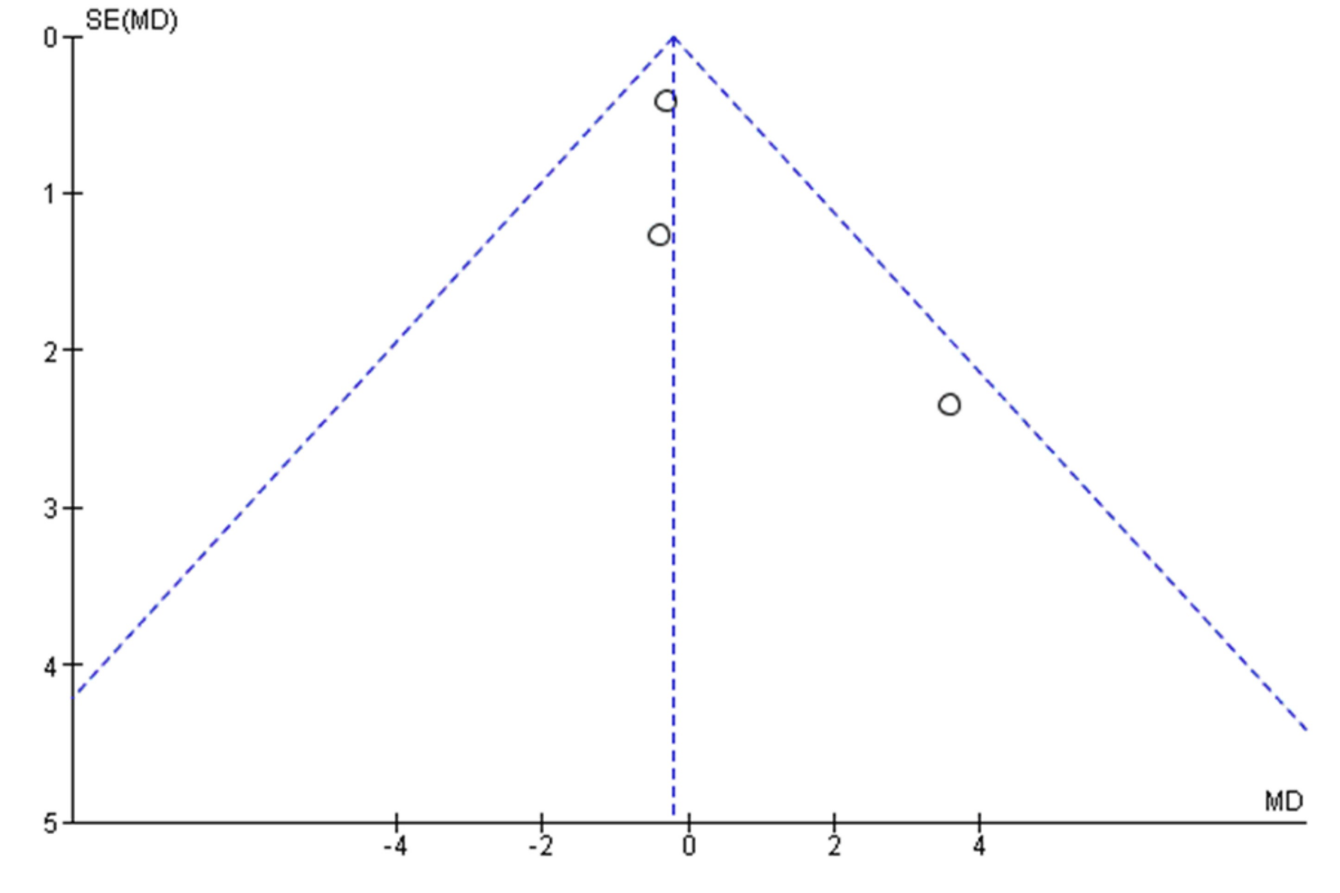
Funnel plot for publication bias: effect of the intergenerational bonding program on the social health outcome SE: standard error; MD: mean difference

The funnel plot evaluating the MD in social health scores (Figure 4) showed no marked asymmetry, indicating no significant evidence of publication bias in the meta-analysis of intergenerational bonding programs' effects on social health.

Outcome Variables

**Figure 5:**
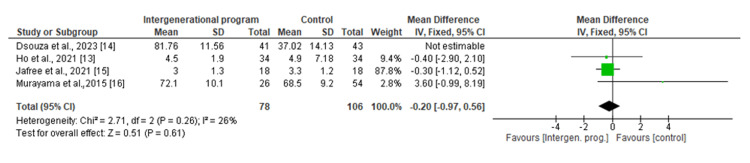
Forest plot of comparison: effect of the intergenerational bonding program on social health CI: confidence interval; SD: standard deviation

Effect of the intergenerational bonding program on social health: The total pooled result from the two studies [13,15] showed a significant improvement in the mean social health score in the intergenerational program group as compared to the control group with an MD of -0.27 (95% CI -0.48 to -0.06; p=0.02*) (Figure 5). A total of 53 participants were in the intergenerational program group, whereas 51 were in the control group. No significant heterogeneity was found in the pooled analysis (I^2^=0%; p=0.34). A sensitivity analysis could not be performed as only two studies were included, with no heterogeneity in the pooled analysis.

**Figure 6:**
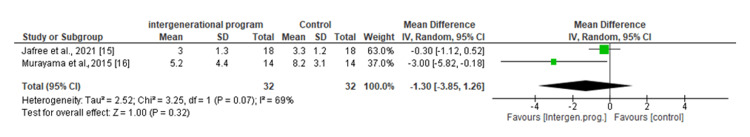
Forest plot of comparison: effect of the intergenerational bonding program on mental health CI: confidence interval; SD: standard deviation

Effect of the intergenerational bonding program on mental health: The total pooled result from the three studies [13,15,16] showed a non-significant improvement in the mean mental health score in the intergenerational program group as compared to the control group with an MD of -0.10 (95% CI -0.24 to 0.03; p<0.14) (Figure 6). A total of 80 participants were in the intergenerational program group, whereas 105 were in the control group. No significant heterogeneity was found in the pooled analysis (I^2^=0%; p=0.75). A sensitivity analysis could not be performed as there was no heterogeneity found in the pooled analysis of the outcome variables.

**Figure 7:**
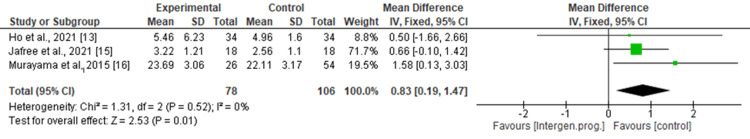
Forest plot of comparison: effect of the intergenerational bonding program on meaningfulness CI: confidence interval; SD: standard deviation

Effect of the intergenerational bonding program on meaningfulness: The total pooled result from the two studies [15,16] showed a significant improvement in the mean meaningfulness score in the control group as compared to the intergenerational program group with an MD of 0.86 (95% CI 0.19 to 1.53; p<0.01*) (Figure 7). A total of 44 participants were in the intergenerational program group, whereas 72 were in the control group. Overall, significant heterogeneity was found to be very low in the pooled analysis (I^2^=18%; p=0.27). A sensitivity analysis was done by omitting Ho et al. [13] to reduce the heterogeneity level in the pooled analysis; hence, Jafree et al. [15] and Murayama et al. [16] were included in the pooled analysis for this endpoint assessment analysis with a low 18% heterogeneity (Table 3).

**Figure 8:**
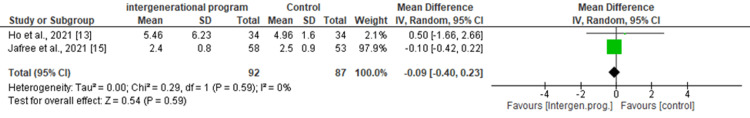
Forest plot of comparison: effect of the intergenerational bonding program on physical health CI: confidence interval; SD: standard deviation

Effect of the intergenerational bonding program on physical health: The total pooled result from the two studies [13,15] showed a non-significant improvement in the mean physical health score in the intergenerational program group as compared to the control group with an MD of -0.03 (95% CI -0.06 to 0.12; p<0.47) (Figure 8). A total of 53 participants were in the intergenerational program group, whereas 51 were in the control group. No significant heterogeneity was found in the pooled analysis (I^2^=0%; p=0.47). A sensitivity analysis could not be performed as only two studies were included with no heterogeneity level in the pooled analysis.

**Figure 9:**
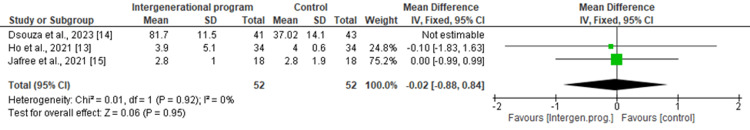
Forest plot of comparison: effect of the intergenerational bonding program on well-being CI: confidence interval; SD: standard deviation

Effect of the intergenerational bonding program on well-being: The total pooled result from the two studies [13,15] showed a significant improvement in the mean well-being status in the control group as compared to the intergenerational program group with an MD of 0.32 (95% CI 0.06 to 0.58; p<0.02*) (Figure 9). A total of 53 participants were in the intergenerational program group, whereas 51 were in the control group. No significant heterogeneity was found in the pooled analysis (I^2^=0%; p=0.94). A sensitivity analysis was done by omitting the Dsouza et al. [14] trial to reduce heterogeneity in the pooled analysis; hence, Ho et al. [13] and Jafree et al. [15] were included for the primary endpoint assessment analysis with 0% heterogeneity (Table 3).

Discussion

This meta-analysis comprises a quantitative evaluation of existing evidence regarding the impact of intergenerational bonding programs on social health, physical health, mental health, well-being, and meaningfulness in older adults.

Based on the meta-analysis findings, the present study comprises significant findings in the social and mental health outcome variables, favoring the intergenerational program group as compared to the control group, without any heterogeneity. However, it was also observed among the well-being and meaningfulness outcome variables of older adults, but the result favored the control group instead of the intergenerational program group, which were all intervened by the intergenerational bonding program. And the reason for such a finding was that many of the previous studies were not systematically structured, lacked a theoretical framework, possessed heterogeneous data, and had intricacy in the intergenerational bonding program, which tightly constrains intellectual activities of older adults, making complex tasks for them, which withdraws their interest from participating in the program [10,17-20].

Impact on Social Health

In the present meta-analysis, there was a significant improvement in the mean social health score in the intergenerational program group as compared to the control group. A similar finding was supported by two previous studies, where an intergenerational bonding program influences social relationships, imposing an impact on older adults' health. People's social networks tend to get smaller as they get older, which has a negative influence on their health [21,22].

Impact on Mental Health

The present meta-analysis findings indicated improvement in the mean mental health scores among older adults who underwent an intergenerational bonding program, although it was not significant. These findings are supported by previous studies, which also reported no significant improvement in depression score among the experimental group, who all received an intergenerational bonding program [23,24].

A study in rural China found that, after adjusting for confounders and other support types, intergenerational emotional support was significantly associated with increased depressive symptoms in older adults [25]. Furthermore, on the evaluation of the Research of Productivity by Intergenerational Sympathy (REPRINTS) program in Japan, there is a significant elevation of depressive mood among older adults because of increased sense of meaningfulness and sense of manageability among them, but due to the smaller sample size, the generalizability of the study findings is constrained [16]. A study conducted by Najafzadeh Shavaki et al., in the East of Guilan, reported a significant reduction in depression among older adults who underwent a role-playing teaching program [26].

Impact on Meaningfulness

In positive psychology, a meaningful life means a life with purpose, significance, fulfilment, and satisfaction. A review identified human relationships as a key factor influencing the sense of meaning in life among older adults [27]. In the present study, there is a significant improvement in the mean meaningfulness score in the control group as compared to the intergenerational program group. In contrast, a Japanese study using the REPRINTS program, where older adults read picture books with children, reported a significant rise in their sense of meaningfulness compared to controls [16].

Impact on Physical Health

In the present study, the total pooled result from the studies showed a non-significant improvement in the mean physical health score of the intergenerational program group as compared to the control group. On the contrary, a review on the impacts of intergenerational engagement on older adults' cognitive and health outcomes found that four out of eight studies found significant intergenerational engagement effects on cognitive outcomes and 21 out of 31 on health-related outcomes [10].

Impact on Well-Being

The present meta-analysis showed a significant improvement in the mean well-being status of older adults in the control group. And the reason for such findings can be an intricate set of activities guided by policy, like numerous reading programs in different schools, which can decrease the involvement of older adults in such programs [10]. On the contrary, two previous studies, a quasi-experimental study conducted on intergenerational communication and older adults' well-being, showed a significant improvement in the experimental group, indicating that intergenerational interaction effectively promotes well-being in them [14,28-30].

Strengths and Limitations of the Study

This meta-analysis includes only interventional studies without any heterogeneity for evaluating the impact of an intergenerational bonding intervention on various domains of older adults. This meta-analysis exclusively included quantitative studies that showed an effect of intergenerational bonding programs on social health, mental health, well-being, physical health, and meaningfulness outcome variables among older adults.

One limitation of the meta-analysis is that our searches were strictly limited to research work published in the English language. We also included only online published studies and left out the grey literature, which could have resulted in publication bias affecting study results.

## Conclusions

The primary outcome of the social health of older adults who underwent an intergenerational bonding program was found to be significantly improved as compared to the control group. Social health, in a long way, can decrease the mortality rate among them. The study findings support a strong recommendation for the implementation of the intergenerational program to uplift the social relationships of older adults.

Additionally, mental health outcomes favor the intergenerational program group, but it was not statistically significant with no heterogeneity, which requires more RCTs on this outcome for better clarity in results.

Although the occurrence of ambiguous results for well-being and meaningfulness variables favors the control group, it might be due to the small sample size of the study samples among the included RCTs, intricacy among interventions, and widely used standardized intergenerational models for their implementation.

Thus, there is an utmost need to develop a widely accepted and culturally driven standardized intergenerational bonding model which can overcome the problem of intricacy and can be widely implemented to drive out the potential benefits of an intergenerational bonding program on various domains of older adults. 

## Appendices

**Table 4:** Search strategy in detail (PRISMA 2020 guidelines) PRISMA: Preferred Reporting Items for Systematic Reviews and Meta-Analyses

S. N.	Steps	No. of studies
1	Identification of studies from databases	302
2	Removed for duplicate studies	15
3	Screening of studies	287
4	Excluded (abstract screened, not eligible studies)	273
5	Eligible studies-free full text	14
6	Excluded due to the measurement of outcome not being the same	10
7	Included final studies for this meta-analysis for all five outcomes	4
8	Studies included in the social health outcome	2 out of 4
9	Studies included in the physical health outcome	2 out of 4
10	Studies included in the well-being outcome	2 out of 4
11	Studies included in the meaningfulness outcome	2 out of 4
12	Studies included in the mental health outcome	3 out of 4

PubMed: 52

("Intergenerational Relations"[MeSH Major Topic] AND ("old"[All Fields] AND ("agrosyst geosci environ"[Journal] OR "age"[Journal] OR "age omaha"[Journal] OR "age dordr"[Journal] OR "adv genet eng"[Journal] OR "age"[All Fields]))) AND ((ffrft[Filter]) AND (fha[Filter]) AND (fft[Filter]) AND (2015:2023[pdat]))

Google Scholar: 37

Impact of the Intergenerational Program on Social Relationships among the elderly population [filter-2014-2023]

Impact of intergenerational program on health among elderly population [filter-2014-2023]

Impact of the intergenerational program on well-being among the elderly population [filter-2014-2023]

Impact of the intergenerational program on depression among the elderly population [filter-2014-2023]

Impact of intergenerational program on meaningfulness among the elderly population [filter-2014-2023]

Scopus: 57

TITLE-ABS-KEY (Intergenerational AND elderly AND health AND social AND well-being) AND PUBYEAR > 2014 AND PUBYEAR < 2024 AND ( LIMIT-TO ( DOCTYPE , "ar" ) ) AND ( LIMIT-TO ( LANGUAGE , "English" ) )

ScienceDirect: 156

Term(s): Intergenerational bonding program AND old age AND health AND well-being AND social not children
